# Serum amyloid A‐to‐albumin ratio as a potential biomarker to predict the activity, severity, and poor prognosis of systemic lupus erythematosus

**DOI:** 10.1002/jcla.24282

**Published:** 2022-02-10

**Authors:** Liang Zhao, Qun Zhang, Zhigang Feng, Jinshan Zhang, Feng He

**Affiliations:** ^1^ Department of Laboratory Medicine The Traditional Chinese Medicine Hospital of Taihe Fuyang China; ^2^ Department of Rheumatology and Immunology, The Traditional Chinese Medicine Hospital of Taihe Fuyang China; ^3^ Department of Laboratory Medicine and Blood Transfusion Zhuzhou Hospital Affiliated to Xiangya Medical College of Central South University Zhuzhou China

**Keywords:** serum albumin, serum amyloid A, SLE disease activity index, systemic lupus erythematosus

## Abstract

**Objectives:**

To evaluate the predictive value of serum amyloid A‐to‐albumin ratio (SAR) for active systemic lupus erythematosus (SLE), severe active SLE, and poor prognosis of SLE.

**Methods:**

One hundred and eighty‐six patients with SLE undergoing treatment in our hospital were selected. The demographic characteristics, clinical data, and disease prognosis of all patients were collected and analyzed.

**Results:**

There were significant differences in SLEDAI, total glyceride (TG), serum amyloid A (SAA), SAR, urinary microalbumin‐to‐creatinine ratio (ACR), erythrocyte sedimentation rate (ESR), albumin (ALB), complement 3 (C3), anti‐dsDNA, anti‐Sm positive rate, and anti‐dsDNA positive rate between active SLE and stable SLE patients. TG, SAR, C3, ACR, and positive anti‐dsDNA were independent influencing factors of active SLE, and the odds ratio (OR) values were 2.342, 10.921, 0.832, 1.451, and 2.476, respectively. The area under curves (AUCs) of SAA, ALB, and SAR for predicting active SLE and severe active SLE were 0.743, 0.724, 0.787, 0.711, 0.686, and 0.733, respectively. The AUC of SAR for predicting the poor prognosis of active SLE was 0.719. High SAR, high ACR, low C3, and positive anti‐dsDNA were high risk factors for poor prognosis. Kaplan–Meier (K‐M) survival analysis showed that patients with high SAR, high ACR, low C3, and positive anti‐dsDNA had shorter continuous remission time than that with low SAR, low ACR, high C3, and negative anti‐dsDNA.

**Conclusion:**

SAR had high predictive value for active SLE, severe active SLE, and poor prognosis of SLE. High SAR may be a potential marker for predicting the activity and prognosis of Chinese patients with SLE.

## Graphical abstract

Inflammatory cytokines play an important role in the pathogenesis and disease progression of SLE. C‐reactive protein‐to‐serum albumin (CAR), as a new inflammatory marker, has been proved to have good predictive value for the diagnosis or prognosis of RA and SLE, and so on, but there is no report on the predictive value of SAR for active SLE, severe active SLE, and poor prognosis of SLE. Our study revealed that TG, SAR, C3, ACR, and positive anti‐dsDNA were independent influencing factors of active SLE. SAR had the highest predictive value for active SLE and severe active SLE and was an independent risk factor for poor prognosis of active SLE. High SAR had significantly shorter continuous remission time than patients with low SAR, which may be a potential marker for predicting the activity and prognosis of Chinese patients with SLE.

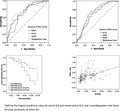

## 1 INTRODUCTION

Systemic lupus erythematosus (SLE) is a chronic, inflammatory, and autoimmune disease,^1^ which damages multiple organs and tissues, including the kidney, liver, and nervous system.^2^, ^3^According to previous studies, SLE patients with high disease activity index have more active immune systems and more disordered inflammatory indexes, and as a result, they suffer from more serious tissues damage.^4^ Previous studies also showed that the disease activity indexes are closely related to the types and doses of hormones^5^; hence, timely and effective evaluation of activity indexes is very important in disease treatments. At present, the SLE Disease Activity Index (SLEDAI) and the British Isles Lupus Assessment Group Index^6^, ^7^ which mainly depend on laboratory test indexes and clinical symptom indexes are commonly used to evaluate disease progression and the prognosis of patients with SLE. However, both scoring methods are complex and time‐consuming.

An important clinical feature of autoimmune diseases is the disorder of inflammatory factors, such as serum amyloid A (SAA), C‐reactive protein (CRP), albumin (ALB), erythrocyte sedimentation rate (ESR), and neutrophil‐to‐lymphocyte ratio,^8^, ^9^ which have been proved to be related with the severity and prognosis of the disease, and the predictive value of different indicators are different. Hwang YG et al.^10^ showed that with the increase of disease activity in rheumatoid arthritis (RA), the levels of CRP and SAA increased, but SAA could better respond to this trend. Shen C et al.^11^ showed that compared with CRP, SAA can better reflect the disease activity score for 28 joints (DAS28) of RA, and the correlation index with DAS28 was higher than that of CRP.

The ideal predictor of disease activity should have high degree of sensitivity and specificity and have certain predictive value for the severity and prognosis of the disease; in addition, the detection process needs to be simple and fast. Serum amyloid A (SAA), as an acute phase response protein, is widely used in the diagnosis of infectious diseases and the evaluation of the therapeutic effect.^12^ Wang CM et al.^13^ found that SAA was not only positively correlated with SLEDAI, but also an independent influencing factor of active SLE. Yip J et al.^14^ found that ALB in patients with active SLE was significantly lower than that in stable SLE, and ALB was negatively correlated with SLEDAI. C‐reactive protein‐to‐serum albumin (CAR), as a new inflammatory marker, has been proved to have good predictive value for the diagnosis or prognosis of RA^15^ and SLE,^16^ and so on. At present, there is no report on the predictive value of SAR for active SLE, severe active SLE, and poor prognosis of SLE. This study compared the predictive value of SAA, serum albumin (ALB) and SAR for active SLE, and severe active SLE, in order to provide a new predictive biomarker for the disease activity and prognosis.

## 2 MATERIALS AND METHODS

### 2.1 Study population

This is a prospective study on the predictive value of SAR in active SLE, severe active SLE, and poor prognosis of SLE. SLE patients diagnosed in Traditional Chinese Medicine Hospital of Taihe from January 2018 to March 2020 were included in this study. All patients included must be over 18 years old and met the 1997 diagnostic criteria of the American College of Rheumatology.^17^ Patients diagnosed with RA, tumors, pregnancy, infectious diseases, liver hepatitis, steatosis, cirrhosis, and other diseases that can affect SAR were excluded. Patients who had been treated systematically before admission were also excluded. Mild active SLE was treated with low‐dose hormone (bonasone acetate) and hydroxychloroquine sulfate. Moderate active SLE was treated with medium dose hormone (bonasone acetate), hydroxychloroquine sulfate, and mild immunotherapeutic agents (methotrexate, leflunomide, and azathioprine). Severe active SLE was treated with high‐dose hormone (necessary shock dose) and cyclophosphamide or mycophenolate mofetil. After discharge, the dosage of hormone was gradually reduced in patients with mild and moderate active SLE, and the induction treatment scheme for severe active SLE was transformed into maintenance treatment scheme. All patients were regularly evaluated (monthly) after discharge, so as to adjust the treatment schemes in time. This study was approved by Ethics Committee of Traditional Chinese Medicine Hospital of Taihe, and all patients signed the agreement.

### 2.2 Data extraction

A total of 22 laboratory indicators, including liver and kidney function, blood lipid, autoantibody, and immunological test results, were collected in this study. The detection of clinical biochemistry indexes [e.g., aspartate aminotransferase (AST), alanine aminotransferase (ALT), total cholesterol (TC), triglyceride (TG), SAA, ALB, and urinary microalbumin‐to‐creatinine ratio (ACR)] and immunological indexes [e.g., Complement 3 (C3), Complement 4 (C4), immunoglobulin M (IgM), immunoglobulin A (IgA), and immunoglobulin G (IgG)] were measured by Hitachi automatic biochemical analyzer (Japan), and the detection of auto antibody spectrum [e.g., anti‐double stranded DNA (anti‐dsDNA), anti‐SjÖgren syndrome A antigen (anti‐SSA), anti‐SjÖgren syndrome B antigen (anti‐SSB), anti‐Sm, anti‐nuclear antibody (ANA), anti‐nucleosome, anti‐histone, and anti‐U1RNP] and anti‐dsDNA by ELISA were measured by HUMAN‐IMTEC (Germany).

### 2.3 Definition

#### 2.3.1 Active SLE and severe active SLE

According to the SLEDAI table, 0–4 points were defined as stable SLE (64 patients), 5–9 points were defined as mild active SLE (43 patients), 10–14 points were defined as moderately active SLE (18 patients), and ≥15 points were defined as severe active SLE (61 patients). Active SLE which included mild, moderately, and severe active SLE was defined as SLEDAI ≥4 points.

#### 2.3.2 Prognosis of SLE

The prognosis of patients was divided into clinical remission group and poor prognosis group. Clinical remission which includes complete remission, clinical hormone‐free remission, and clinical hormonal remission is defined as SLEDAI ≤4. (1) Complete remission is defined as SLEDAI = 0, and anti‐malarial drugs can be used. (2) Clinical hormone‐free remission is defined as clinical static, serologically active, stable clinical presentation, SLEDAI ≤4, allowing the use of antimalarials and immunosuppressive drugs. (3) Clinical hormonal remission is defined as the use of antimalarials and immunosuppressants drugs in serologically active, clinically stationary patients who take hormone less than 5 mg per day.^18^ Poor prognosis includes disease recurrence or death. Disease recurrence is defined as the increase of SLEDAI ≥3 after stabilization, or patients develop new skin and oral ulcer, serositis, arthritis, fever, central nervous system changes, vasculitis, nephritis, myositis, platelet count ≤60 × 10^9^/L, hemolytic anemia (hemoglobin <70g/L), or patients need to strengthen immunosuppressive treatment and hospitalization.^19^

### 2.4 Statistical analysis

Spss20.0 was performed to establish a database. Categorical variables were presented as counts and compared by chi‐squared test. Normal distribution data were presented as the mean ± standard deviation (SD) and compared with Student's *t*‐test. Non‐normal distribution data were presented as the median and interquartile range (IQR) and compared with Mann–Whitney U‐test. Binary logistic regression analysis was used to find the independent influencing factors for active SLE, and Spearman's correlation analysis was used to analyze the correlation between two continuous variables. The receiver operating curve (ROC) was used to analyze the predictive value of different indicators for active SLE, severe active SLE, and poor prognosis of SLE, and the optimal clinical cutoff value was determined by the maximum Youden index (Youden index =sensitivity+ specificity‐ 1). Medcalc software was used for area under curve (AUC) comparison, and the Z‐test was used to compare the predictive ability of different indicators. Potential risk factors which identified in a univariate model were included in a multivariate model. Kaplan–Meier (K‐M) analysis was used to estimate the survival curve of sustained remission between different groups, and log‐rank test was used to analyze the differences between two groups. *p* < 0.05 means the difference was statistically significant.

## 3 RESULT

### 3.1 Clinical characteristics of participants

**FIGURE 1:**
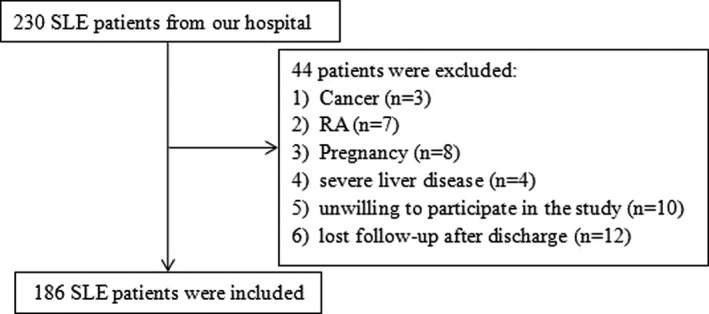
Flow chart illustrating the inclusion and exclusion criteria of our study cohorts

From January 2018 to March 2020, 230 patients were diagnosed with SLE in Traditional Chinese Medicine Hospital of Taihe. Among them, 3 patients were with cancer, 7 patients were with RA, 8 patients were with pregnancy, 4 patients were with severe liver disease, 10 patients were unwilling to participate in the study, and 12 patients lost follow‐up after discharge, above of them were excluded from the study. Finally, 186 SLE patients were included in the study (Figure 1). Table 1 shows the clinical characteristics of all participants. The average age of participants was 38.05 years old, ranging from 20 to 56, and the ratio of male to female was 1: 9.94. There was no significant difference in age, sex, and disease duration between active SLE and stable SLE.

**TABLE 1:** Results of univariate and multivariate analysis predicting active SLE

Variable	Univariate analysis			Multivariate analysis		
	Active SLE (*n* = 122)	stable SLE (n = 64)	*p*	OR	95%CI	*p*
Age (years)	38.33 ± 8.52	37.52 ± 7.94	0.529			
Sex (male/female)	11/111	6/58	0.936			
Disease duration (months)	24.72 ± 10.12	22.51 ± 11.04	0.172			
SLEDAI	13(9–22)	3(1–3)	0.000			
AST (U/L)	38.92 ± 10.92	39.62 ± 9.67	0.667			
ALT (U/L)	42.51 ± 11.34	41.36 ± 12.51	0.527			
TG (mmol /L)	2.69 ± 0.65	2.43 ± 0.58	0.008	2.342	1.196~5.124	0.039
TC (mmol /L)	5.05 (4.62–5.37)	4.89 (4.58–5.21)	0.304			
SAA (mg/L)	18.96 ± 9.52	14.33 ± 10.41	0.003	*		
ALB (g/L)	37.59 ± 8.19	41.22 ± 8.03	0.004	*		
SAR (mg/g)	0.51 ± 0.14	0.36 ± 0.18	0.000	10.921	7.654~24.117	0.019
C3 (g/L)	0.62(0.47–0.73)	0.72(0.55–0.89)	0.006	0.832	0.641~0.963	0.028
C4 (g/L)	0.12 ± 0.10	0.14 ± 0.09	0.182			
IgA (g/L)	2.62 ± 0.78	2.51 ± 0.65	0.335			
IgG (g/L)	17.42 ± 4.47	16.82 ± 3.96	0.367			
IgM (g/L)	1.27 ± 0.62	1.32 ± 0.58	0.594			
ACR (mg/g)	64.34 ± 36.51	38.95 ± 20.12	0.000	1.451	1.076~4.610	0.031
ESR (mm/h)	36.52 ± 10.12	27.54 ± 9.77	0.000	*		
Anti‐dsDNA (IU/ml)	62.41(24.21–124.32)	38.57(18.62–66.33)	0.000	*		
Anti‐dsDNA (+)	76(62.29)	25(39.06)	0.003	2.476	1.384~4.721	0.007
Anti‐SSA (+)	78(63.93)	36(56.25)	0.307			
Anti‐SSB (+)	22(18.03)	11(17.19)	0.886			
Anti‐Sm (+)	47(38.52)	15(23.44)	0.038	1.616	0.830~3.144	0.156
Anti‐ANA (+)	122(100.00)	64(100.00)	1.000			
Anti‐ nucleosome (+)	31(25.40)	15(23.44)	0.767			
Anti‐histone (+)	22(18.03)	11(17.19)	0.886			
Anti U1‐RNP(+)	51(41.80)	25(39.06)	0.718			
ACA(+)	81(66.39)	44(68.75)	0.745			

Abbreviations: ACA, Anti‐cardiolipin antibody; ACR, urinary microalbumin‐to‐creatinine ratio; ALB, serum albumin; ALT, alanine aminotransferase; ANA, Anti‐nuclear antibody; anti‐SSA, anti‐SjÖgren syndrome A antigen; Anti‐SSB: anti‐SjÖgren syndrome B antigen; AST, aspartate aminotransferase; C3, complement 3; C4, complement 4; ds‐DNA, double stranded DNA; ESR, erythrocyte sedimentation rate; IgA, immunoglobulin A; IgG, immunoglobulin G; IgM, immunoglobulin M; SAA, serum amyloid A; SAR, serum amyloid A‐to‐serum albumin; SLEDAI, SLE disease activity index; TC, total cholesterol; TG, triglyceride.

*Variables were not included in the equation.

### 3.2 Analysis on influencing factors of active SLE

All SLE patients were divided into active SLE group and stable SLE group according to whether SLEDAI ≥5. Testing the baseline clinical data between two groups, 11 factors shown differed significantly (Table 1). High level of SLEDAI, TG, SAA, SAR, ACR, ESR, anti‐dsDNA, anti‐Sm positive rate, and anti‐dsDNA positive rate and low level of ALB and C3 were observed in active SLE, and the differences between the two groups were significant. In order to ensure the stability of the model, all factors closely related to SAR, including SAA, ALB, and ESR, were excluded. All potential predictors, including TG, SAR, C3, ACR, positive anti‐dsDNA, and anti‐Sm, were further analyzed by binary logistic regression analysis. In the multivariate analysis, we found that TG, SAR, ACR, and positive anti‐dsDNA were independent influencing factors of active SLE, while C3 was protective factor of active SLE, and the OR values were 2.342, 10.921, 1.451, 2.476, and 0.832, respectively (*p *< 0.05), but positive anti‐Sm was not an independent factor of active SLE (*p *> 0.05).

### 3.3 Predictive value of SAA, ALB, and SAR for active SLE

**FIGURE 2:**
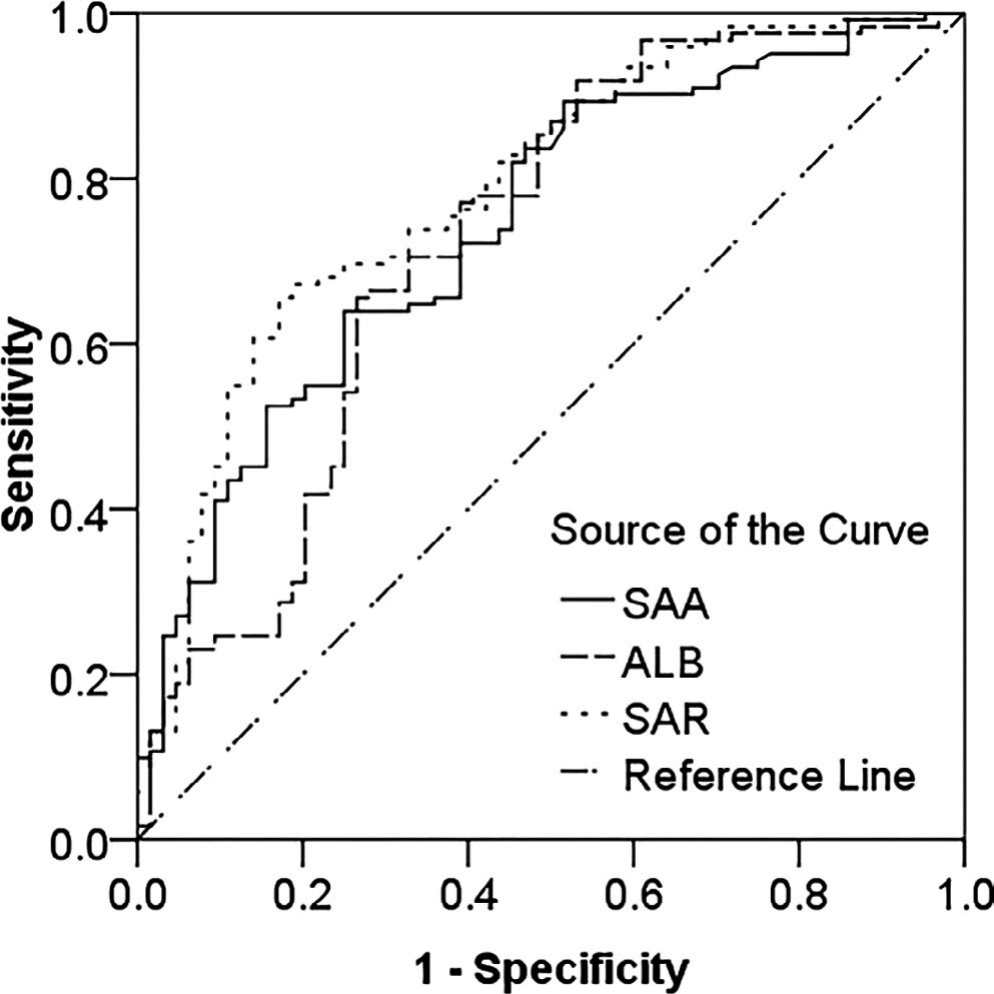
ROC curve analysis of SAA, ALB, and SAR to distinguish active SLE from stable SLE

The AUCs of SAR, SAA, and ALB for predicting active SLE were 0.787, 0.743, and 0.724, respectively. Compared with SAA and ALB, SAR had the highest predictive value. The optimal cutoff value of SAR was 0.43 mg/g, and the prediction sensitivity and specificity were 67.20% and 79.70%, respectively. The optimal cutoff value of SAA was 16.05 mg/L, and the prediction sensitivity and specificity were 63.90% and 75.00%, respectively. The optimal cutoff value of ALB was 38.50 g/L, and the prediction sensitivity and specificity were 66.40% and 71.90%, respectively (Figure 2).

### 3.4 Predictive value of SAA, ALB, and SAR for severe active SLE

**FIGURE 3:**
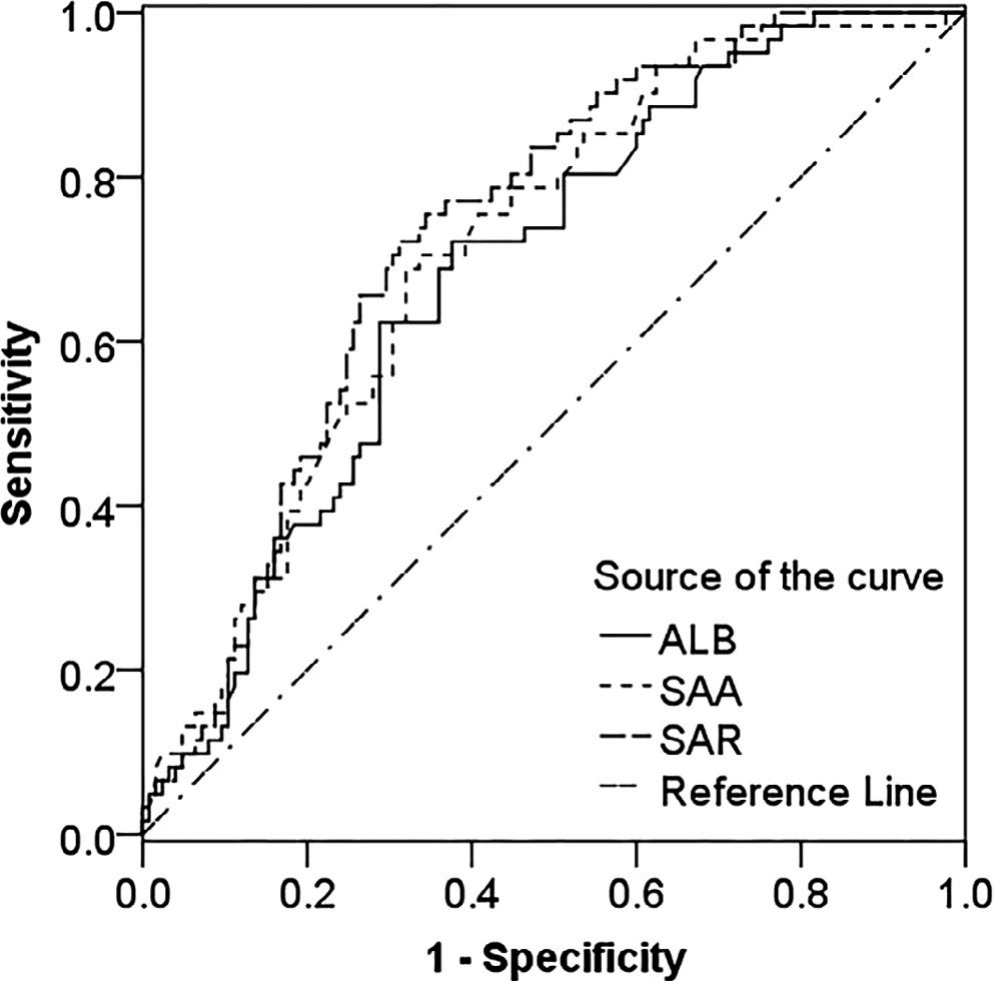
ROC curve analysis of SAA, ALB, and SAR to identify patients with severe active SLE

The AUCs of SAR, SAA, and ALB for predicting severe active SLE were 0.733, 0.711, and 0.686, respectively. Compared with SAA and ALB, SAR had the highest predictive value. The optimal cutoff value of SAR was 0.51 mg/g, and the prediction sensitivity and specificity were 75.40% and 65.60%, respectively. The optimal cutoff value of SAA was 19.15 mg/L, and the prediction sensitivity and specificity were 68.90% and 68.00%, respectively. The optimal cutoff value of ALB was 36.70 g/L, and the prediction sensitivity and specificity were 72.10% and 62.40%, respectively. (Figure 3).

### 3.5 Correlation analysis between SAA, ALB, SAR, and SLEDAI

**FIGURE 4:**
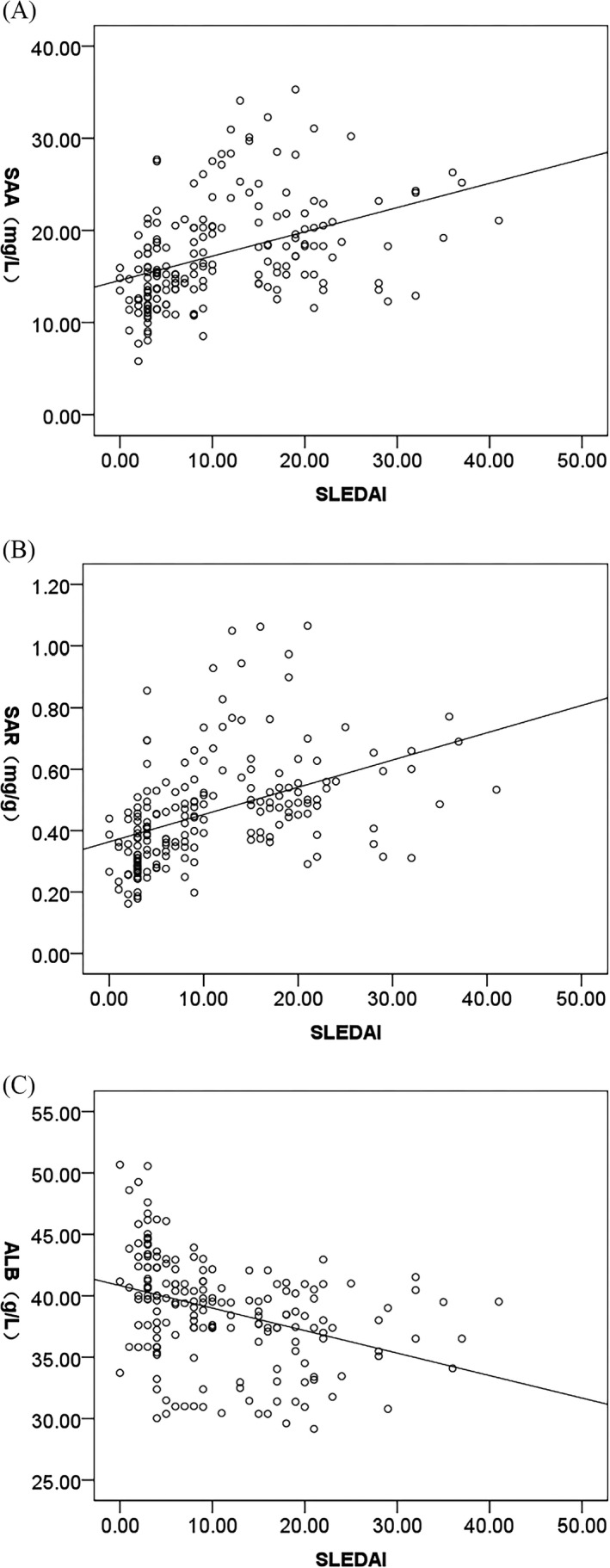
Correlation analysis between SAA, ALB, SAR, and SLEDAI

The correlation analysis results showed that SLEDAI was significantly and positively correlated with SAA (Figure 4A, *r* = 0.409, *p *= 0.000), but negatively correlated with ALB (Figure 4C, *r* = −0.368, *p *= 0.000). A stronger positive correlation was observed between SAR and SLEDAI (Figure 4B, *r* = 0.440, *p *= 0.000).

### 3.6 Risk factor of poor prognosis in active SLE

During the 15‐month observation period after disease remission, 43 of the 122 patients with active SLE had a poor prognosis. On the univariable Cox regression analyses, we found higher TG [hazard ratio, HR = 3.745 95%CI: (1.168~8.782), *p *= 0.036], higher SAA [HR = 1.098, 95%CI: (1.050~1.149), *p* = 0.015], lower ALB [HR = 0.958, 95%CI: (0.915~0.997), *p* = 0.038], higher SAR [HR = 11.497, 95%CI: (2.973~44.461), *p* = 0.000], lower C3 [HR = 0.901, 95%CI: (0.844~0.952), *p* = 0.015], higher ACR [HR = 1.024, 95%CI: (1.011~1.892), *p* = 0.042], higher ESR [HR = 1.235, 95%CI: (1.004~1.669), *p* = 0.024], higher anti‐dsDNA [HR = 1.162, 95%CI: (1.073~2.054), *p* = 0.039], and positive anti‐dsDNA [HR = 1.959, 95%CI: (1.082~3.549), *p* = 0.015] were associated with an increased probability of poor prognosis. There were five variables which were selected for multivariate analysis using a forward step wise method. In the multiple Cox regression, we found higher SAR [HR = 7.956, 95%CI: (1.772~36.312), *p* = 0.010], lower C3 [HR = 0.821, 95%CI: (0.786~0.951), *p* = 0.008], higher ACR [HR = 1.471, 95%CI: (1.224~1.757), *p* = 0.012], and positive anti‐dsDNA [HR = 2.017, 95%CI: (1.196~4.021), *p* = 0.025] were independently associated with high risk of poor prognosis. Obviously, high level of SAR was associated with the greatest hazard for poor prognosis (Table 2).

**TABLE 2:** Results of Cox regression predicting poor prognosis in active SLE patients

Variables	The univariable Cox regression analyses		The multiple Cox regression analyses	
	HR(95%CI)	*p* value	HR(95%CI)	*p* value
Age (years)	1.442 (0.821~2.069)	0.249		
Sex (male/ female)	1.424 (0.873~2.975)	0.341		
Duration (months)	1.394 (0.920~1.968)	0.189		
SLEDAI	1.037 (0.998~1.078)	0.066		
AST (U/L)	1.103 (0.842~1.996)	0.301		
ALT (U/L)	1.098 (0.832~2.012)	0.414		
TG (mmol /L)	3.745 (1.168~8.782)	0.036	2.476(0.982~8.366)	0.230
TC (mmol /L)	1.024 (0.968~1.352)	0.121		
SAA (mg/L)	1.098 (1.050~1.149)	0.015	*	
ALB (g/L)	0.958 (0.915~0.997)	0.038	*	
SAR (mg/g)	11.497 (2.973~44.461)	0.000	7.956(1.772~36.312)	0.010
C3 (g/L)	0.901 (0.844~0.952)	0.015	0.821(0.786~0.951)	0.008
C4 (g/L)	0.942 (0.876~1.032)	0.064		
IgA (g/L)	0.997 (0.932~1.127)	0.137		
IgG (g/L)	1.072 (0.964~2.510)	0.364		
IgM (g/L)	1.212 (0.974~3.542)	0.401		
ACR (mg/g)	1.024 (1.011~1.892)	0.042	1.471(1.224~1.757)	0.012
ESR (mm/h)	1.235 (1.004~1.669)	0.024	*	
Anti‐dsDNA (IU/ml)	1.162 (1.073~2.054)	0.039	*	
Anti‐dsDNA (+)	1.959 (1.082~3.549)	0.015	2.017(1.196~4.021)	0.025
Anti‐SSA (+)	1.468(0.682~3.158)	0.325		
Anti‐SSB (+)	1.057(0.881~1.268)	0.539		
Anti‐Sm (+)	1.090(0.507~2.344)	0.826		
Anti‐ nucleosome (+)	0.818(0.352~1.900)	0.640		
Anti‐histone (+)	1.057(0.881~1.268)	0.539		
Anti‐U1RNP(+)	1.136(0.816~1.581)	0.437		
ACA(+)	1.172(0.681~2.018)	0.561		

Abbreviations: ACA, Anti‐cardiolipin antibody; ACR, urinary microalbumin‐to‐creatinine ratio; ALB, serum albumin; ALT, alanine aminotransferase; ANA, Anti‐nuclear antibody; anti‐SSA, anti‐SjÖgren syndrome A antigen; Anti‐SSA, anti‐SjÖgren syndrome B antigen; AST, aspartate aminotransferase; C3, complement 3; C4, complement 4; ds‐DNA, double stranded DNA; ESR, erythrocyte sedimentation rate; IgA, immunoglobulin A; IgG, immunoglobulin G; IgM, immunoglobulin M; SAA, serum amyloid A; SAR, serum amyloid A‐to‐serum albumin; SLEDAI, SLE disease activity index; TC, total cholesterol; TG, triglyceride.

*Variables were not included in the equation.

### 3.7 Analysis of prognostic differences among different groups

**FIGURE 5:**
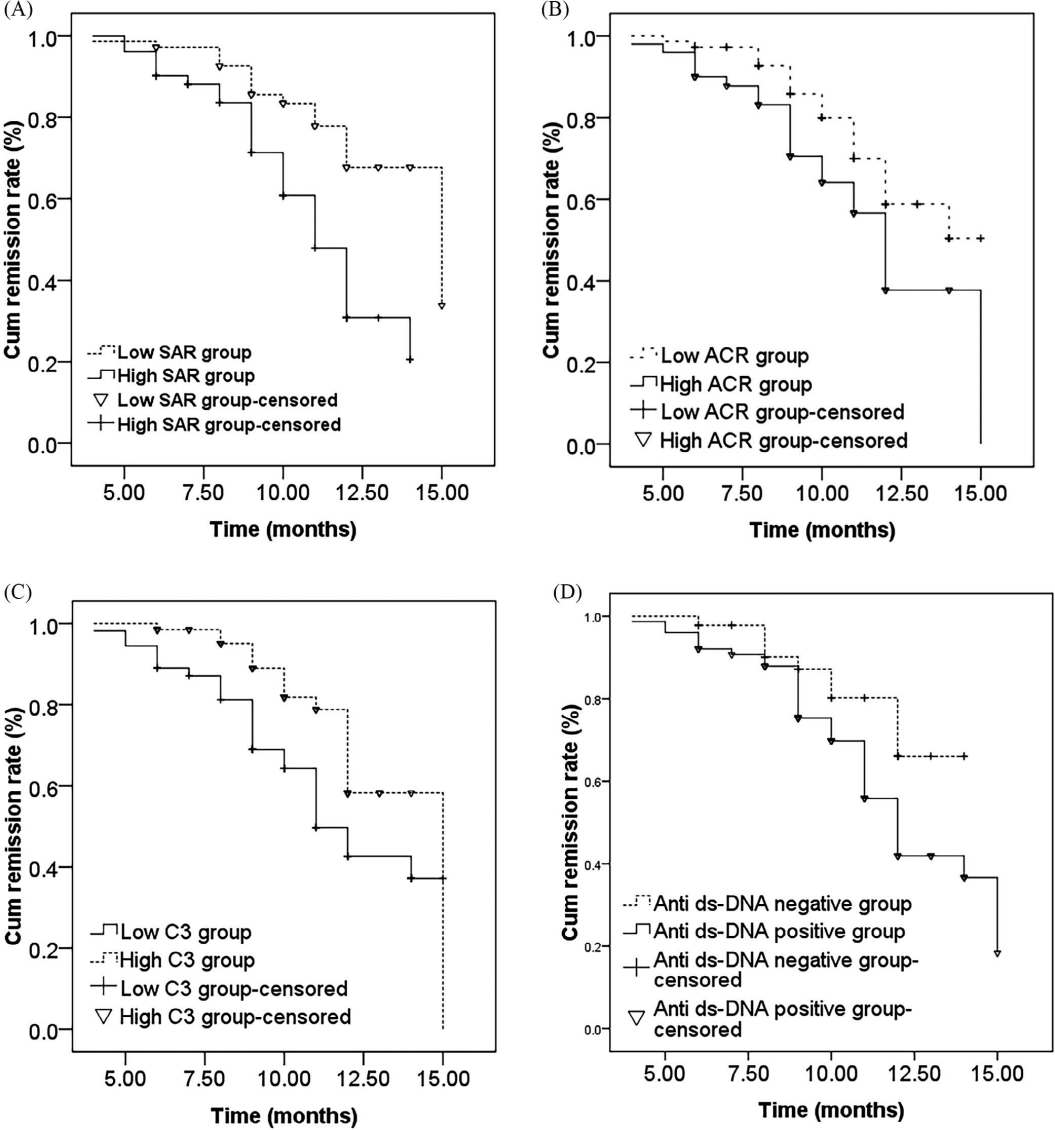
K‐M analysis for poor prognosis in active SLE patients with different levels of SAR, ACR, C3, and anti‐dsDNA

According to the mean values of SAR, C3, ACR, and the results of anti‐dsDNA, patients with active SLE were divided into high SAR group, low SAR group, high C3 group, low C3 group, high ACR group, low ACR group, anti‐dsDNA positive group, and anti‐dsDNA negative group. K‐M survival curve was used to estimate continuous remission over time in different groups, and the results showed patients with high SAR (SAR≥0.51mg/g) or low C3 (C3≤ 0.62 g/L) had significantly shorter continuous remission time (*p* < 0.001) than patients with low SAR (SAR<0.51 mg/g, Figure 5A) or high C3 (C3>0.62 g/L, Figure 5C). It also showed patients with high ACR (ACR≥64.34 mg/g) or positive anti‐dsDNA were more like to have shorter continuous remission time than patients with low ACR (ACR<64.34 mg/g, Figure 5B) or negative anti‐dsDNA (Figure 5D), and the differences were statistically significant.

### 3.8 Predictive value of SAR for poor prognosis in active SLE

**FIGURE 6:**
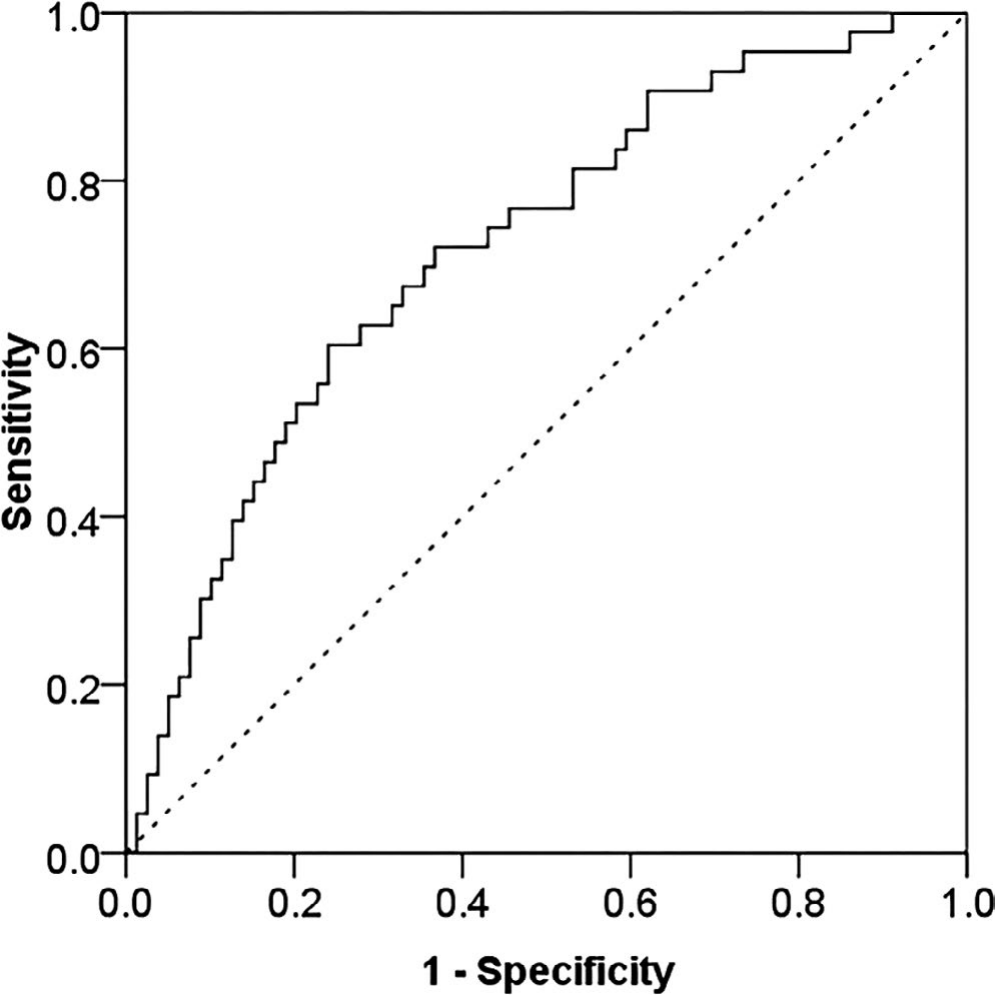
ROC curve analysis of SAR for poor prognosis in active SLE

ROC curve analysis revealed that the AUC of SAR was 0.719, the optimal cutoff value was 0.53 mg/g, and the prediction sensitivity and specificity were 72.10% and 63.30%, respectively (Figure 6).

## 4 DISCUSSION

To the best of our knowledge, this is, thus far, the first study for SAR in SLE. The purpose of this study was to investigate the predictive value of SAR in active SLE, severe active SLE, and poor prognosis of SLE. Our findings showed that SAR had high predictive value for active SLE, severe active SLE, and poor prognosis of SLE. High SAR, high ACR, high TG, low C3, and positive anti‐dsDNA were demonstrated to be independent influence factors for active SLE. In addition, high SAR, high ACR, low C3, and positive anti‐dsDNA were demonstrated to be independent prognostic factors for shorter continuous remission time in patients with active SLE. High SAR shown great associated with an increased probability of poor prognosis in our follow‐up time, it means SAR would be a good potential marker of predicting disease severity and prognosis in SLE patients.

SLEDAI score includes 24 indexes such as leukocyte count, complement, hematuria, and proteinuria,^20^ including both laboratory test indexes and clinical symptom indexes, which is inconvenient for clinical operation. The rapid, sensitive, and specific evaluation of disease activity for patients with SLE is important for both short‐term and long‐term treatment planning.^21^ Previous studies showed that inflammatory factors, such as tumor necrosis factor‐α, C3, C4, erythrocyte distribution width, platelet to lymphocyte ratio, and neutrophil‐to‐lymphocyte ratio,^22^, ^23^, ^24^ were related to the severity of SLE. These studies support that inflammatory cytokines play an important role in the pathogenesis and disease progression of SLE. ALB which is an important component of serum protein can reflect nutritional status and systemic inflammatory response.^25^ The liver which can induce immune tolerance is the target organ of immune‐mediated damage,^26^ so it can be damaged in SLE, resulting in the reducing of albumin synthesis. Furthermore, the deposition of immune complexes can cause lupus nephropathy, resulting in the increasing protein loss through kidney.^27^ In fact, hypoalbuminemia can often be observed in SLE patients. Anti‐dsDNA as an important index in the evaluation of disease activity of SLE is frequently found both in serum and inflammatory lesions in glomerulonephritis.^28^ The fact that circulating antibody levels are usually associated with active SLE and renal involvement has strengthened the assumption of pathogenetic importance of anti‐dsDNA.^29^ In our study, we found that SAR, C3, TG ACR, and anti‐dsDNA were independent influencing factors of active SLE. In addition, SAA and SAR were positively correlated with SLEDAI, but ALB was negatively correlated with SLEDAI. These three indicators had high predictive value for active SLE and severe active SLE, and the predictive value of SAR was significantly higher than SAA and ALB. The reasons may be that SAR combines the positive correlation factor and the negative correlation factor, which expand the inflammatory difference between active SLE and stable SLE, severe active SLE, and non‐severe active SLE. Therefore, SAR prediction value was obviously higher than a single index.

Many studies proved that laboratory indicators not only had important reference value for SLE diagnosis, but also could judge the activity, recurrence, and treatment effect, including age, sex, race, economic, and organ damage.^30^ According to previous research, the prognosis of Caucasians was better than Blacks,^31^ the prognosis of men was worse than women,^32^ and the prognosis of patients with superior family economic conditions and high educational level was better than patients with poor economic conditions and low educational level.^33^ However, there are few studies on which laboratory indexes and clinical symptoms related to the prognosis of SLE. Feng XN reported that the decreasing of CD4 ^+^ T lymphocytes and the increasing of ESR were risk factors for poor prognosis.^34^ Pang J revealed that the level of C5a in SLE may be a marker of prognostic judgment.^35^ In our study, we found that the poor prognosis rate of active SLE was 35.25%, which was higher than that reported above. The reason may be related to the longer follow‐up of this study than above. We also found that the increasing of SAR, ACR, the decreasing of C3, and the positive of anti‐dsDNA were risk factors for poor prognosis in active SLE. K‐M survival analysis further showed that patients with high SAR, high ACR, low C3, and positive anti‐dsDNA had shorter continuous remission time than that with low SAR, low ACR, high C3, and negative anti‐dsDNA. The above results were reported for the first time.

However, this study had several limitations. Firstly, there was a small sample size included in this study, resulting in obvious break points of ROC curve analysis. Secondly, the influence of treatment factors on active SLE after discharge was not analyzed. In the future, multicenter, big data and multi‐index prospective research may help us further understand the influencing factors of pathogenesis and treatment.

## 5 CONCLUSION

This study aimed to investigate the predictive value of SAR for active SLE, severe active SLE, and poor prognosis of SLE. Our data indicated that high SAR may be a potential biomarker for predicting the activity and poor prognosis of Chinese patients with SLE. This potential indicator can help doctors predict the disease severity and prognosis of SLE in time. SAA and ALB should be detected for such patients, and the treatment plan should be adjusted according to SAR to prevent further progress of the disease.

## CONFLICT OF INTEREST

The authors declare that there is no conflict of interest.

## Data Availability

The data are available upon reasonable request.
